# A Comparative Study of Electric Load Curve Changes in an Urban Low-Voltage Substation in Spain during the Economic Crisis (2008–2013)

**DOI:** 10.1155/2014/948361

**Published:** 2014-04-15

**Authors:** Pedro M. Lara-Santillán, Montserrat Mendoza-Villena, L. Alfredo Fernández-Jiménez, Mario Mañana-Canteli

**Affiliations:** ^1^Department of Electrical Engineering, University of La Rioja, 26004 Logroño, Spain; ^2^Department of Electrical and Energy Engineering, University of Cantabria, 39005 Santander, Spain

## Abstract

This paper presents a comparative study of the electricity consumption (EC) in an urban low-voltage substation before and during the economic crisis (2008–2013). This low-voltage substation supplies electric power to near 400 users. The EC was measured for an 11-year period (2002–2012) with a sampling time of 1 minute. The study described in the paper consists of detecting the changes produced in the load curves of this substation along the time due to changes in the behaviour of consumers. The EC was compared using representative curves per time period (precrisis and crisis). These representative curves were obtained after a computational process, which was based on a search for days with similar curves to the curve of a determined (base) date. This similitude was assessed by the proximity on the calendar, day of the week, daylight time, and outdoor temperature. The last selection parameter was the error between the nearest neighbour curves and the base date curve. The obtained representative curves were linearized to determine changes in their structure (maximum and minimum consumption values, duration of the daily time slot, etc.). The results primarily indicate an increase in the EC in the night slot during the summer months in the crisis period.

## 1. Introduction

Currently, the knowledge of the load curve is considered a fundamental factor to ensure the precise and accurate operation of power systems. In this field, short- [1], mid- [2], and long-term [3] demand forecasting has been investigated. To realise this forecasting, the load curves in previous years are studied. Very short-time prediction, on the order of a few minutes, may soon be needed because of the emergence of new electric loads (as the electric vehicles) that can suppose a change in the philosophy of the use of electric systems [4].

Electric load curve studies have been considered for decades. This curve determines multiple aspects of planning [5–8], the control and operation of electric power systems [9–11], and the calculation of costs associated with electricity distribution and the sharing of these costs [12, 13]. The electric load curve must be adjusted to the electric energy generation curve to avoid power quality problems due to the wave quality and supply reliability. Therefore, several research groups have studied consumption curves to foresee the power supplies or the design of various electrical infrastructures associated with a service zone. Some groups have attempted to forecast the peak values [6, 8, 14–20] or average values [21, 22] to design the infrastructures or their support equipment.

In some studies, the authors made assumptions based on aggregate criteria [10, 23–26]. In these works, the total consumption, associated with the electricity supply point, was assumed to correspond to the weighted sum of different consumers associated with that connection point. This assumption was based on the known change in consumption of each of the consumer groups, which depends on consumer behaviour. Other researchers have worked to identify different groups of consumers and presented demand disaggregation-based methods [27, 28]. Artificial intelligence techniques have also been applied to load curve forecasting [29, 30]. Various tools have been used in these approaches, such as the clustering of standard curves and the study of the dependence on external factors, mainly weather variables. The data are usually clustered based on the types of consumers or days (day of the week, season of the year, etc.). As such, Seem [31] performed a clustering analysis to determine the days of the week on which the energy consumption was similar in three buildings.

Several factors as the size of study area, location of countries, and cadence of measurements records are used as contrast methods. The size of the study area varies greatly. Previous studies of load curves have covered small sectors of a city (even at the level of a single building [32]), one or more cities [33], a country [24], or a set of countries [34]. The cadence of the measurements records typically takes values between 15 minutes [10, 35] and one hour [19]. In studies focused purely on energy, the time between successive records may be one month [36]. In economic studies, the electricity consumption (EC) is usually measured annually [37].

Blázquez et al. [38] proposed an empirical analysis of various characteristics of Spanish residential electricity demand. They paid special attention to the influence of electricity price, country income, and weather conditions on the electricity demand. As a result, they found that weather variables significantly impacted the demand when accounting for the outdoor temperature. Similarly, many studies have investigated the influence of the temperature on the electric load curve [39, 40]. The models proposed by Kavousian et al. [41] showed that the daily minimum EC was mainly influenced by weather, location, and physical characteristics of the building and the daily maximum EC was influenced by end uses that were energy intensive and did not run constantly. This group of end uses mostly depended on the occupancy levels and activities of occupants.

For our study, we measured the EC with a sampling time of 1 minute during a period of 11 years (2002–2012). The measuring instrument (LEM TOPAS 1000) was installed in an urban low-voltage substation of the city of Logroño (placed in the north of Spain). The maximum, average, and minimum temperatures on days under study were recorded (*t*_max⁡_, *t*_av_, *t*_min⁡_) for the same time period. We considered the electrical measurements of all dates for the study eliminating atypical dates (source of outliers). These atypical dates included general strikes and mass sporting events. Furthermore, dates in which some type of error was produced, either of measurements or of the electricity supply, were also eliminated. Each of the remaining dates was taken as a “base date” for the search for comparable days depending on the temperature, calendar date (15 days before and after the base date in all measurement years), the day of the week (working day or not), and the daylight time. Finally, the comparable dates were clustered to a certain base date by establishing a separation between the dates belonging to the economic crisis period (from the last months of 2007) and those prior to this event.

The global financial crisis, started in 2007, strongly influenced euro area economies and for some countries—notably Ireland and Spain—it triggered a banking crisis as real estate prices started to fall. These banking crises in turn contributed to the sovereign debt crises in these two countries [42]. We used the end of 2007 as the beginning of the economic crisis in Spain since it was the moment when the first signs appeared as a high deficit in the regional governments and a decline in the economic activity [43].

In the study described in this paper, which is detailed in [44], we develop a new methodology for the comparison of load curves taking into account a large number of parameters that may influence the EC. This methodology involves several stages: filtering, denoising, clustering, and linearization All these stages help to simplify the comparison process between the EC load curves belonging to the two periods (precrisis and crisis). The obtained results in this comparison lead to the identification of slight differences in the EC curves in the crisis period with respect to those in the precrisis period.

The paper is structured as follows: Section 2 presents the methodology used in the work described in the paper; Section 3 describes the case study: the application of the proposed methodology to the load curves obtained from an urban low-voltage substation; Section 4 includes and discusses the results obtained in the case study; lastly, the conclusions are presented in Section 5.

## 2. Description of the Proposed Methodology

**Figure 1:**
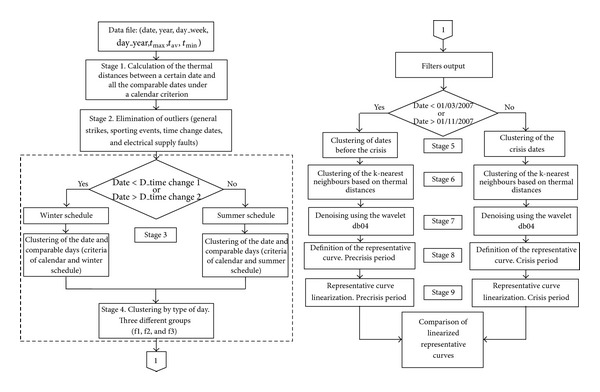
Stages of the proposed methodology.

Figure 1 defines the stages of the methodology followed for the analysis of the changes in the electric load curves during the economic crisis (2008–2013).

### 2.1. First Stage: Calculation of the Thermal Distances between a Certain Date and All the Comparable Dates under a Calendar Criterion

Initially, a text file with the structure defined in Table 1 was built. In this file, the indexing field was the measurement date, which was broken down into a second field, that is, the year. In addition to the daily temperatures previously mentioned (average, minimum, and minimum outdoor temperatures), the other variables stored in the file were the day of the week and day of the year (1–365 or 1–366). All the temperatures were expressed in degrees Celsius.

The day of the week corresponded to a computer code: 1 = Sunday, 2 = Monday,…, 7 = Saturday, except on the public holidays, marked with 11, and the regional or local holidays (in Logroño, one week around September 21 and five days around June 11), to which 20 was added, that is, 21, 22,…, 27. Table 1 shows a small fragment of the temperature file.

Using the data from this file, each date that serves as the base date was logically crossed with comparable dates that were close on the calendar. In each crossing, it calculated the thermal distance between the daily temperature vector of the base date and that of the comparable date. For the purposes of the calendar, the base date was compared with the 14 days previous to and following this date over the 11 years of measurements.

The daily temperature vector of a date *i* was represented by *T*_*i*_ = (*t*_max⁡ *i*_, *t*_av *i*_, *t*_min⁡ *i*_), where *t*_max⁡ *i*_, *t*_av *i*_, and *t*_min⁡ *i*_ correspond to the maximum, average, and minimum outdoor temperatures for the date *i*. Equation (1) represents the thermal distance between the temperatures of the day *i* and day *j*, which was calculated with the Euclidian distance

(1) d(Ti,Tj) =(tmax⁡i−tmax⁡j)2+(tav i−tav j)2+(tmin⁡i−tmin⁡j)2.

Therefore, the result of the first stage was a data file with the thermal distances between a given date (base date) and the dates that might be comparable by their proximity on the calendar.

### 2.2. Second Stage: Elimination of Outliers

In the 11 years corresponding to the measurements of EC in the low-voltage substation (on a minute basis) and temperature (on a daily basis), occurred various incidents that might influence the electric load curve. The thermal distances of the crosses between comparable dates, in which one of the two dates corresponded to one of these events, were not calculated (assigned value: −1). The considered incidents are summarised below.

- Anomalies, such as voltage drops or supply problems, and measurement errors.
- Days on which the legal time changes (daylight saving time), which implies a movement of one hour in the sunlight time. These days are special because they have a different number of hours than the remaining days (23 or 25 hours); therefore, they are not comparable to any other day of the year.
- Any other date that incurs doubts about the validity of the measurements (general strikes and sporting events have a significant impact).

An outlier detection technique based on the classification of objects that can be identified with labels was used. In our case, the data labels were dates. The result of this stage was a file that contained the thermal distances of the crossings between the base date and the comparable dates according to their proximity on the calendar with the elimination of the dates that presented any incident, which were considered as outliers. The distance value was −1 when one of the dates that were compared had been defined as outlier.

### 2.3. Third Stage: Clustering by the Time Change Criterion

At this stage, we separated the dates by the solar time criterion. To properly compare two days of the year, they must belong to the same solar time, which is defined by the two annual time changes. The days of year were divided into two categories: winter time and summer time. The legal time changes in Spain are made in March (summer time) and October (winter time).

The next step involved the generation of clusters, which were formed by the base date and other dates comparable under a calendar criterion and belonging to the same category according to the solar time. This stage produced a set of clusters equal in number to the measurement days.

### 2.4. Fourth Stage: Clustering by the Day Type

The behaviour of consumers with regard to the EC depends on the day of the week [45]. The electric load curve varies substantially from a working day to a holiday or a day on weekend. Furthermore, the EC differs between workdays and Fridays. The behaviour of consumers on Saturday is not comparable to that one of any other day of the week. Therefore, the clusters can be combined in various ways by selecting different groupings of days to compare EC values. Therefore, the data were clustered based on the day type in this stage.

Three types of associations were considered.

- Filter f1. C_1_: working days (Mondays to Fridays), C_2_: Saturdays, and C_3_: Sundays + Holidays.
- Filter f2. C_1_: Mondays to Thursdays, C_2_: Fridays, C_3_: Saturdays, and C_4_: Sundays + Holidays.
- Filter f3. C_1_: Mondays, C_2_: Tuesdays, C_3_: Wednesdays, C_4_: Thursdays, C_5_: Fridays, C_6_: Saturdays, and C_7_: Sundays + Holidays.

The public and regional or local holidays were included in the same cluster (as holidays). The application of the weekday criterion to the clusters obtained in the previous stage generated a new file. Each filter generated a unique file that provided a value of −1 to the thermal distance of the days that were not in the same cluster of the base date. The following stages (from fifth to ninth) were applied to the base date cluster formed from grouping by type of day.

### 2.5. Fifth Stage: Clustering Precrisis and Crisis Dates

The data were divided into two clusters after this stage. One cluster corresponded to the dates prior to the economic crisis (precrisis) and the other dates belonged to the crisis period. The starting date of the economic crisis varies by source (second semester of 2007 or first semester of 2008). The exact date of the crisis onset is less significant because the electric meter was damaged and a gap in the measurement register was generated during this period (from March to October 2007). In our case, we have considered the crisis period beginning in the last two months of 2007. In this step of the methodology, two clusters were obtained. One of the clusters contained the thermal distances of the dates before crisis and the other contained those during the crisis.

### 2.6. Sixth Stage: Clustering Based on Thermal Distances

Once the previous stages were completed and the clustering methods were applied, the study focused on choosing dates that were more similar to the base date in thermal distance values. Dates with thermal distance to the base date lower than a defined threshold were selected in the cluster defined in the fifth stage. The result of this stage was a couple of clusters of dates per filter (one per period). The couples with the dates which had a minimum of elements in each cluster were stored.

### 2.7. Seventh Stage: Filtering or Denoising the Load Curve

**Figure 2:**
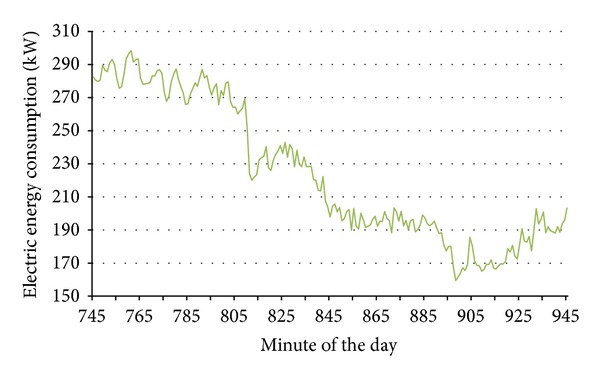
Load curve fragment with a sampling time of 1 minute.

Once comparative dates were selected according to calendar, time change, day type, and thermal distance criteria, we worked with the minute EC curves of these dates. The load curves obtained with a sampling time of 1 minute showed a considerable amount of noise (Figure 2). This noise could negatively affect the comparison between curves. Therefore, filtering these curves was advisable to reduce these abrupt consumption changes and to smooth the curves. By filtering the signal (EC or load value), random events that occur during a measurements day could be eliminated. The selection of filters for smoothing the load curve and reducing the error was not a trivial task. Here, this task was accomplished by wavelet denoising, which presents various advantages compared to other techniques, such as the singular spectrum analysis [46]. To select a specific wavelet family, the use of load curve filtering should be considered to compare minute EC measurements. Thereby, the smoothing of the curve positively correlated with the reduction in errors associated with random phenomena. However, this process incurred a loss of information. We needed to determine a compromise between obtaining fewer errors and appropriate smoothing.

At this stage, the base date EC curve and all the curves belonging to the same cluster were filtered. The selected filtering technique, as it is shown in the case study, was the Daubechies db04 wavelet.

### 2.8. Eighth Stage: Representative Curves Definition

**Figure 3:**
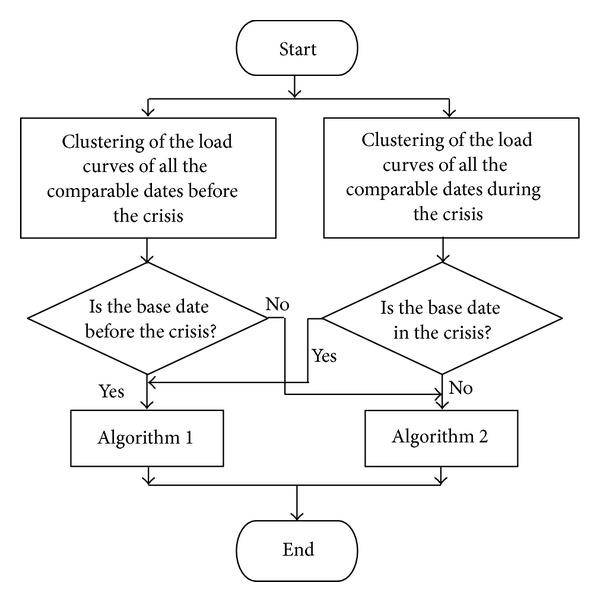
Eighth stage flowchart.

The methodology proposed in this stage is defined in the flowchart of Figure 3. This methodology differs depending on the calendar period that contains the base date (precrisis or crisis). Depending on this period, two different algorithms were used.

- Algorithm 1. Search for the representative load curve in the period where the base date was situated.
- Algorithm 2. Search for the representative load curve in the period where the base date was not situated.

The fundamental difference between the two algorithms is the centroid selection method. The centroid is the central point of the cluster, which can be defined in several ways, such as the mean value or the median of the curves associated with the cluster.

#### 2.8.1. Algorithm  1

When the base date was situated in the same period where the representative curve was going to be calculated, the base date load curve was chosen as the cluster centroid. The root mean square error (RMSE) between the centroid and the rest of cluster curves was calculated as follows:

(2) RMSEi=11440∑j=11440(ECbase datej−ECji)2,

where EC_base  date  *j*_ is the base date EC in the minute *j* and EC_*ji*_ is the EC in the minute *j* of the date *i*.

After calculating the errors, the three load curves with lower RMSE were selected (the three nearest neighbours including the own base date curve). The mean minute EC curve was calculated with the three chosen load curves. The value of the representative minute EC curve for minute *j* is defined by

(3) ECrepresentative  curve  j=13∑i=13ECji.

The index, *j*, runs along the curve; that is, it takes values from 1 to 1440, allowing for the obtaining of the EC representative curve for the base date.

Next, the maximum value of the representative curve was normalised by dividing the representative EC curve by its daily maximum consumption (EC_max⁡⁡*i*_). This process is defined by

(4) ECnormalized representative j=ECrepresentative  curve  jECmax⁡⁡i.

#### 2.8.2. Algorithm  2

This algorithm was applied when the base date was not situated in the period where the representative curve would be calculated. This process was different from that described above. In this case, the centroid minute EC of the cluster was calculated using

(5) ECcentroid j=1n7∑i=1n7ECji,

where EC_centroid *j*_ is the EC of the cluster centroid in the *j*th minute and *n*_7_ is the number of elements (load curves) of the cluster obtained in stage 6 and filtered in stage 7. Once the centroid had been calculated, a process similar to algorithm 1 was followed to obtain the representative curve in this time period.

### 2.9. Ninth Stage: Representative Curve Linearization

**Figure 4:**
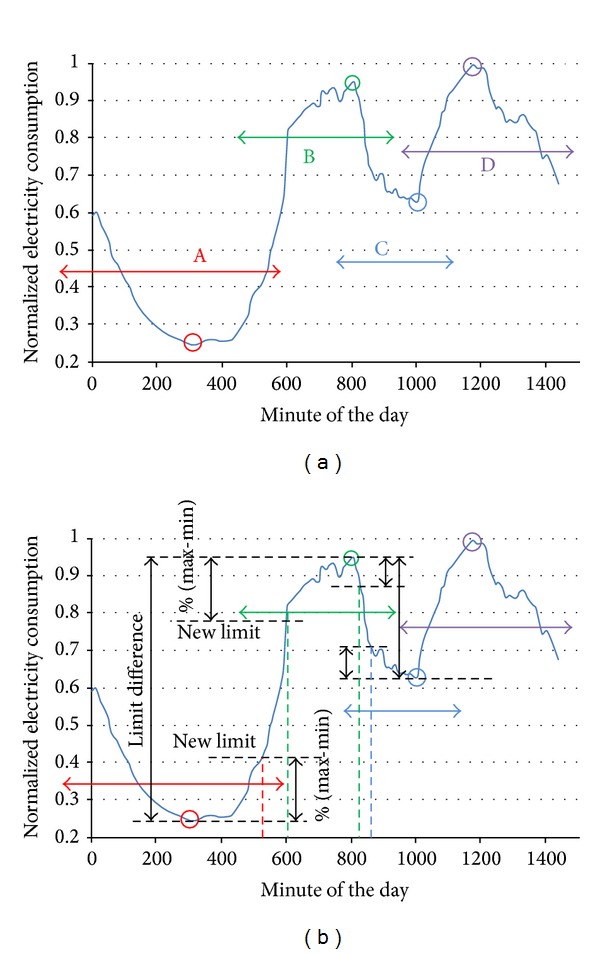
Linearization process. (a) Definition of the first four slots. (b) Definition of limit difference and new limits.

In this stage, the load curves were linearized. Initially, the daily load curve in the linearization process was divided into four periods according to EC levels, as shown in Figure 4(a). These periods were overlapped to avoid losing information in the posterior readjust. These four periods are

- A: from minute 1200 (20:00 h) of the previous day to minute 600 (10:00 h);
- B: from minute 480 (8:00 h) to minute 930 (15:30 h);
- C: from minute 780 (13:00 h) to minute 1140 (19:00 h);
- D: from minute 930 (15:30 h) to minute 1440 (24:00 h).

The extreme values or limits of each defined zone were found (minimum in A and C periods and maximum in B and D periods). These limits are represented in Figure 4(b) with circles. The following step was the recalculation of the time borders associated with the periods. The EC limit values corresponding to the time borders of each period were calculated by taking the difference between maximum and minimum values of two consecutive periods. With that difference, the EC limits of the corresponding period were fixed as the maximum value minus a percentage of the difference for the B and D periods or as the minimum values plus the same percentage of the difference for the A and C periods. The time interval between these new EC limits was considered as an indeterminacy zone; that is, it did not belong to any defined slot. The new borders of the time slots were determined by the crossing of the new consumption limits and the curve.

**Figure 5:**
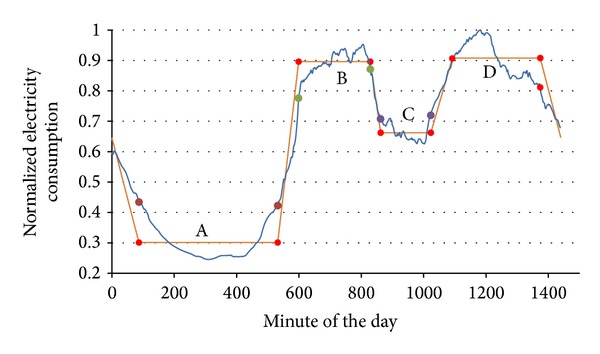
Original and linearized load curves.

Once the borders of the four periods had been calculated, the next step was the determination of the straight lines that defined the EC in each period. Each line was represented by two points. The abscissa of the points was determined by the border value (minute of the day) calculated in the previous step. The ordinate for these points was the mean value of the EC between the calculated border values. The consumption in the time slots was taken constant and had as value the ordinate corresponding to the period.  Figure 5 represents the result of the linearization process for a load curve.

## 3. A Case Study. Obtaining of the Linearized Representative Load Curves of a Specific Date

The EC measurements that were sampled every minute during a period of 11 years (2002–2012) constituted the starting point. The measurements were obtained in an urban low-voltage substation in the city of Logroño, Spain. The number of residential and commercial consumers of this substation was approximately 400; nearly 95% of the customers were residential consumers, and the rest were business consumers (as shops, banks and offices). In the 11 years, the number of customers, and their distribution, did not change significantly. Additionally, the daily outdoor temperatures of the same time period were collected.

### 3.1. Calculation of the Thermal Distances between the Base Date and the Comparable Dates under Calendar Criterion

As mentioned previously in the methodology, this stage ultimately generates a data file that contains the thermal distances between the base date and all comparable dates under calendar criterion. In order to illustrate an example, we have selected February 10, 2005 (Thursday), as base date in this case study. On this day, the registered temperatures were *t*_max⁡_ = 14.5°C, *t*_av_ = 6.5°C, and *t*_min⁡_ = 0.3°C.  Table 2 shows a small fragment of the thermal distances file.

### 3.2. Elimination of Outliers

This stage produced a file that contained the thermal distances between a base date and all the comparable dates under calendar criterion. The outlier dates (those that might contain abnormal EC values) were eliminated from this file. For example, a time change day, such as March 27, 2005 (27/03/2005), constituted an outlier date. The measurement problems were considered as outliers and so they were eliminated (their thermal distances were fixed as −1).

**Figure 6:**
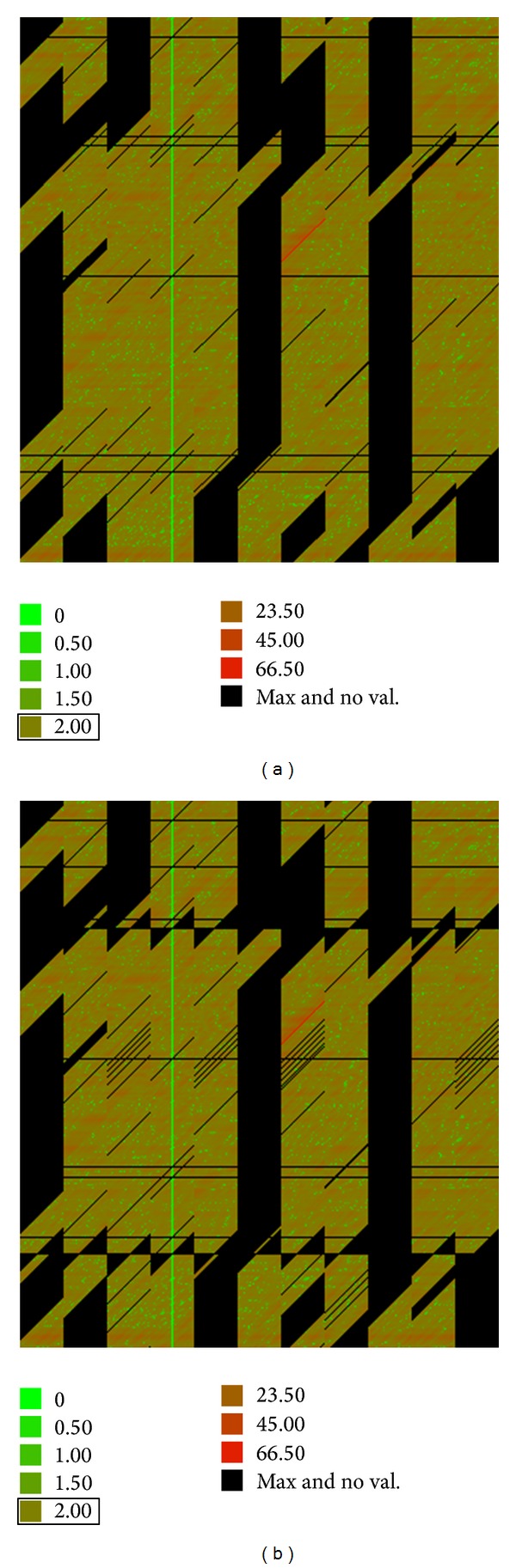
Bitmaps of the thermal distances as a result of the second (a) and third (b) stages.

The thermal distances were represented by a bitmap.  Figure 6(a) shows a bitmap of the thermal distances of the crossings between all days in 2005 (each day is taken as the base date) and their comparable days under calendar criterion. The thermal distances are represented by the range of colours according to the legend. The nonexistent distances (−1) are shown in black. The black horizontal lines correspond to outlier dates.

### 3.3. Clustering by the Time Change Criterion

At this stage, we separated the dates by the solar time criterion. To properly compare two days of the year, they must belong to periods with the same solar time. These periods are limited by the two annual time changes. So the days of year were divided into two categories: those corresponding to the winter time and those corresponding to summer time. The legal time changes in Western Europe take place in March, defined as Date_time change 1 in Figure 1. (starting of summer time), and in October, defined as Date_time change 2 (starting of winter time).

Figure 6(b) shows a bitmap of the thermal distances of the crossings between all the days in 2005 and their comparable days under calendar criterion with the same solar time during 2005. Outliers have been eliminated for these data. The difference between this bitmap and the one of the previous stage (Figure 6(a)) consists of the black teeth that appear above and below the dates of time change. These black areas correspond to dates next to the time change dates. These dates are in different solar time periods, even though they are close on the calendar.

### 3.4. Clustering by the Day Type

**Figure 7:**
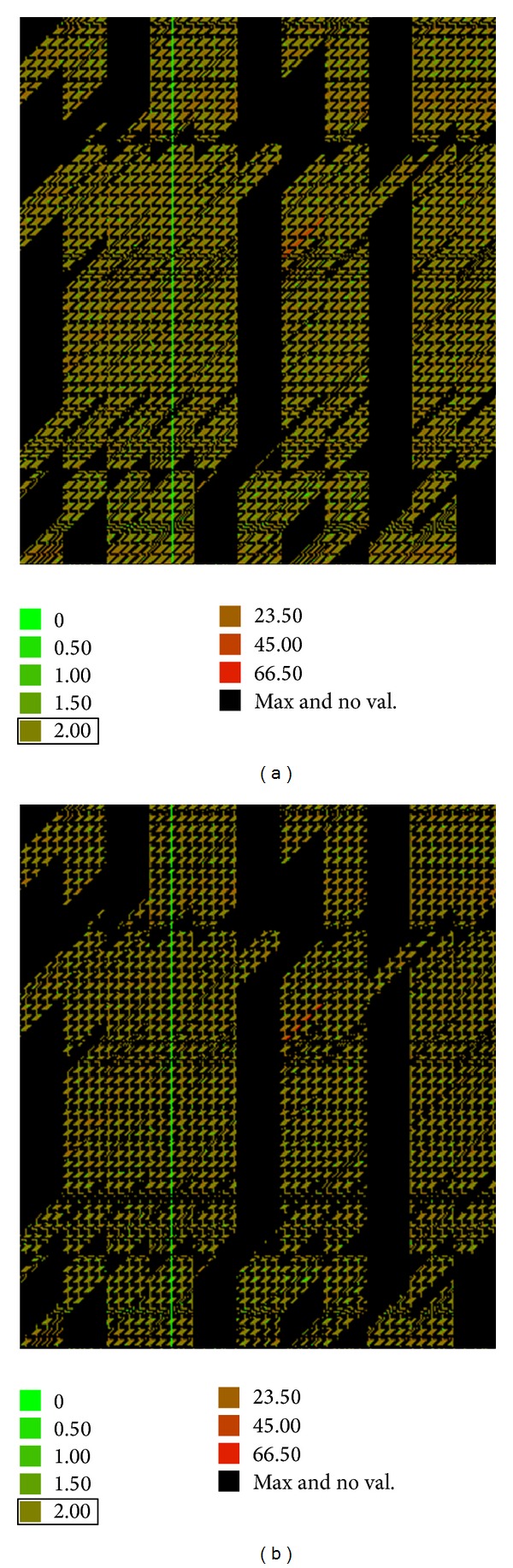
Bitmap of the thermal distances as a result of different filter clustering. (a) f1 and (b) f2.

The developed data files depend on the filter used. These files are also represented by bitmaps, which can be visualised in Figure 7. This result was obtained with filters f1, f2, and f3 during 2005. In these bitmaps, we can verify that increasing the restriction to form clusters (filter f2) reduces the number of available comparable dates. In some cases, the number of dates was insufficient to perform the comparison.

### 3.5. Clustering Precrisis and Crisis Dates

At this point, we had generated a thermal distances file per filter, whose data had passed all previous stages. Now a temporal separation was performed to establish a possible starting date of economic crisis. In our study, we considered the beginning of the crisis in the last months of 2007. This temporal separation was necessary due to the lack of electric measurements from 23/04/2007 to 25/10/2007 as consequence of a fault in the measuring instrument. So each file was divided into two clusters depending on the temporal position with respect to the limit date. One of the clusters contained the thermal distances of the dates before the crisis (before 23/04/2007) and the other contained those values for the dates during the crisis (from 01/11/2007). The data of these two clusters were stored in their respective files. The date selected in this case study is located in the precrisis period.

### 3.6. Clustering Based on Thermal Distances

We studied the effect of the thermal threshold distance on the number of comparable dates. This study was applied to the two sets (couple of clusters) of each filter, one per period. A date was considered comparable when the number of elements in both clusters was equal to or greater than a defined minimum number.

**Figure 8:**
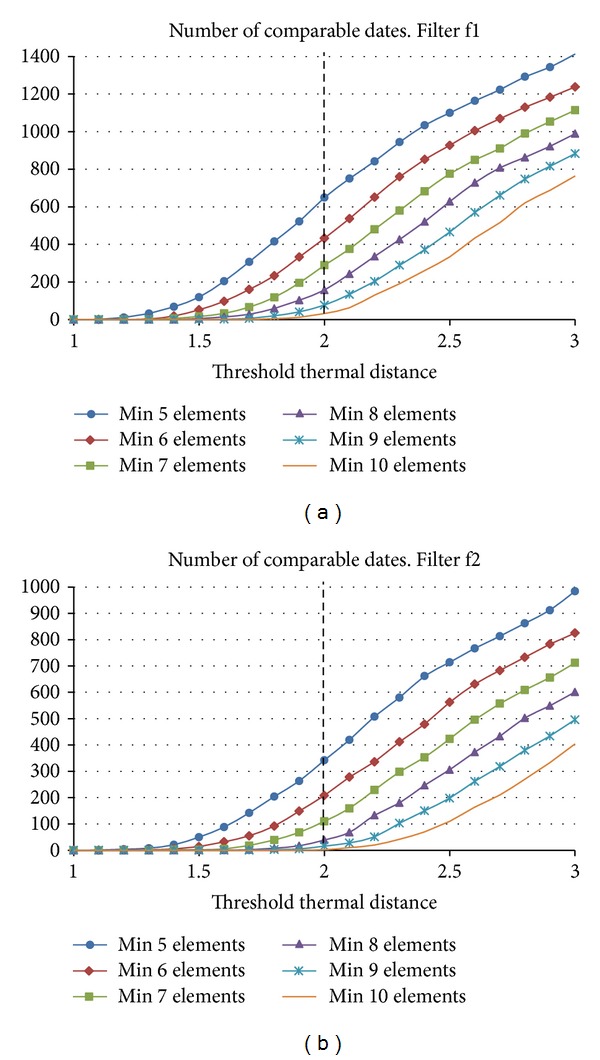
Influence of the minimum number of elements and the threshold thermal distance in the number of comparable dates, once the filters f1 or f2 have been applied.

Figure 8 shows the results of our study. Using more restrictive filters clearly reduced the number of valid comparable dates. Furthermore, if the minimum number of elements increased, the number of obtained comparable dates to a base date was reduced. In order to obtain a sufficient number of comparable dates, we selected 6 elements as a minimum value for this study (the base date curve was considered as one of the elements). We chose a thermal distance threshold of 2°C and a minimum of 6 elements per cluster because they represent a compromise between a representative number of comparable days and a minimum number of elements for choosing among them, in the 8th stage, the nearest neighbours. Based on these conditions, clusters with 645 and 332 comparable dates were obtained for filters f1 and f2, respectively (the base date curve and, at least, other five curves). Filter f3 did not yield a sufficient number of dates.

**Figure 9:**
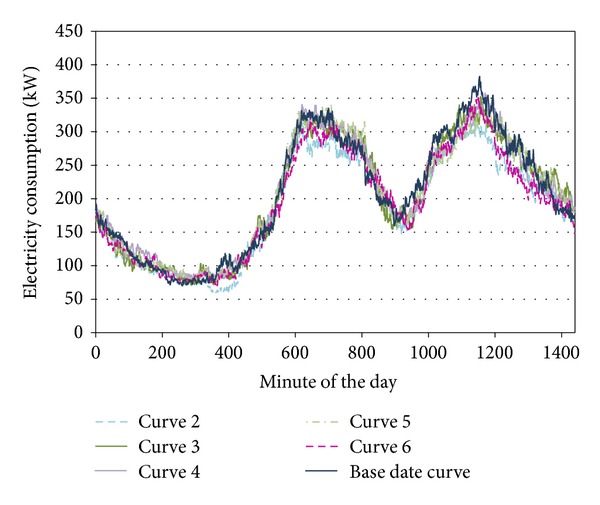
Base date load curve and all curves in cluster corresponding to the precrisis period.

The base date, defined as an example, passed the conditions of filters f1 and f2. Filter f2 was chosen to continue with the example. This date belongs to cluster C1 of this filter (Mondays–Thursdays), and it will only be compared with these weekdays (day type of 2–5). In Figure 9 the load curves obtained in the precrisis period (the base date is 10/02/2005) are represented; there are six load curves, the corresponding to the base date and the load curves in the cluster of that period (5 curves).

### 3.7. Filtering or Denoising of the Load Curve

The 37 mother wavelets used by the Labview software were used in this study; they included the Coiflet, Daubechies, Biorthogonal, and Symlet wavelets. The Daubechies wavelet, db04, was chosen to filter the minute load curves due to its lower error. We determined a balance between a small error and an appropriate smoothing using this wavelet filter.

**Figure 10:**
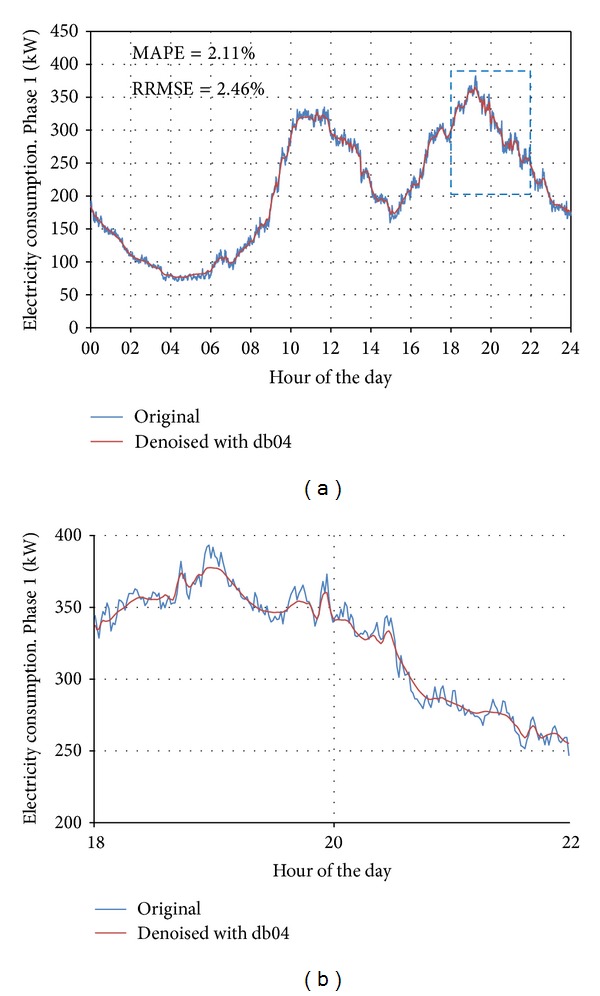
(a) Original and denoised (db04 wavelet) load curves fragment for a Thursday in winter (10/02/2005). (b) Load curves fragment from 18 to 22 hours.

Figure 10(a) shows an example of the representation of the original curve and wavelet db04 filtered load curve.

This figure also includes the denoising error values, the MAPE (mean absolute percentage error) and RRMSE (relative root mean square error), which are defined in (6) and (8). $\overset{-}{\text{EC}}$ represents the daily average value of EC, calculated by (7), and EC_RMS_ represents the root mean square value as follows:

(6) MAPE=(1/1440)∑j=01439|ECj−ECdenoised  j|EC−·100,

(7) $$\begin{array}{c} \overset{-}{\text{EC}}=\frac{\sum_{j=0}^{1439}{\text{EC}}_{i}}{\text{1440}}, \end{array}$$

(8) $$\begin{array}{c} \text{RRMSE}=\frac{\sqrt{\left(\text{1}/\text{1440}\right)\sum_{i=0}^{1439}{\left({\text{EC}}_{j}-{\text{EC}}_{\text{denoised}j}\right)}^{\text{2}}}}{{\text{ EC}}_{\text{RMS}}}\cdot \text{100}. \end{array}$$

The denoised curve presents, with respect to the original one, a MAPE of 2.11% and a RRMSE of 2.46%, denoting that the filtering process has not changed significantly the load curve. In Figure 10(b), a small fragment of both curves is plotted. Notice that, in the denoised curve, the random events have been smoothed. The use of denoised curves simplifies the computational effort in the following stages.

### 3.8. Representative Curve Definition

The curve of the date selected as an example fulfilled the condition established in this study because it had six or more elements (load curves) in each time period. This condition was the first one that was applied at the beginning of the eighth stage to continue with the process.

This date was prior to the crisis. Algorithm 1 was processed to determine the three nearest neighbour curves (included itself as a neighbour) and the representative curve in this time period (precrisis). The base date curve operated as centroid. The three curves were selected among all the elements obtained in the sixth stage (Figure 9) and filtered in the seventh stage. The representative EC curves were calculated using the mean value of the EC of the three curves (minute by minute). The resultant curve of Algorithm 1, which is the representative curve for the selected date in the precrisis period (normalised to a maximum consumption value of 340.5 kW), is shown in Figure 12(a).

**Figure 11:**
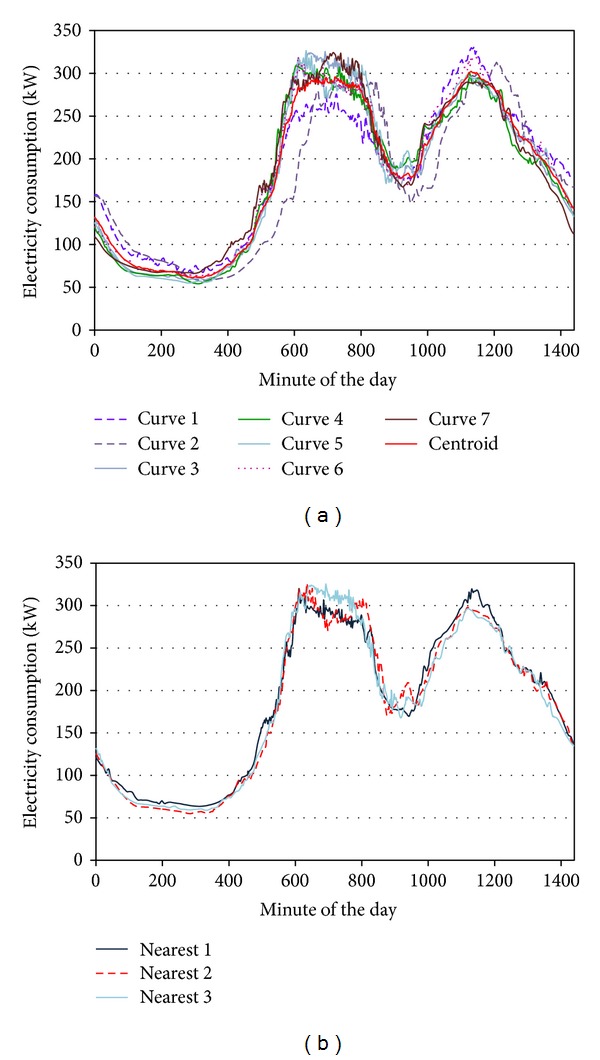
(a) The load curves in the crisis period cluster and centroid of the base date. (b) The three nearest curves during the crisis period.

**Figure 12:**
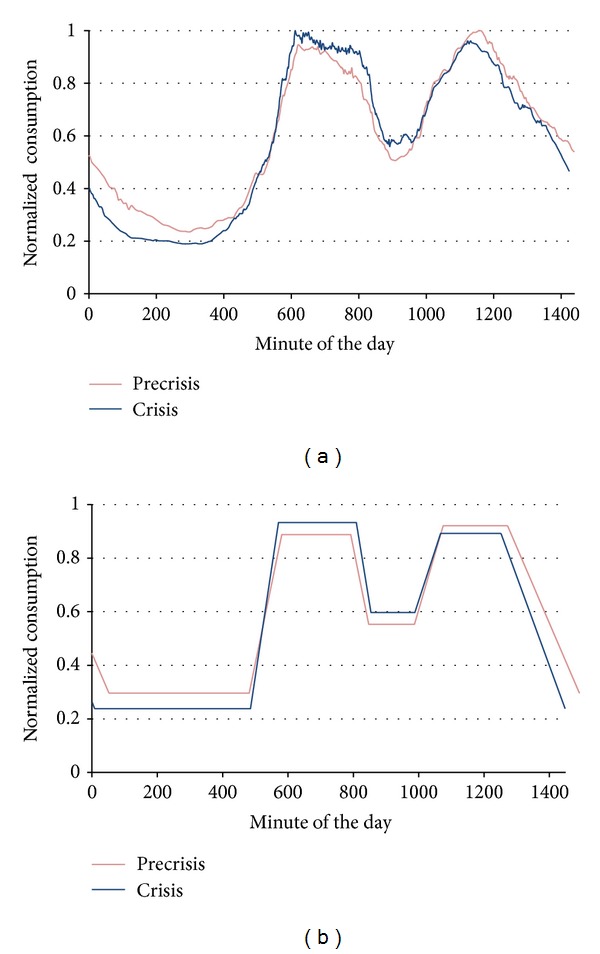
(a) Normalised representative curves of the base date in the two time periods (precrisis and crisis). (b) Linearized representative curves.

Algorithm 2 was executed to obtain the representative curve of the base date during the crisis. The centroid was calculated from the EC values of the curves found in the sixth stage and filtered in the seventh stage.  Figure 11(a) shows the centroid and the nearest curves (there were 7 elements in the corresponding cluster after the sixth stage). The three curves that showed lower errors with respect to the centroid were selected (Figure 11(b)). The representative EC curves in the crisis period were calculated in the same way as those in Algorithm 1, that is, from the mean value of the EC of the three nearest curves and normalised to the maximum consumption value (315.55 kW). This curve is also shown in Figure 12(a).

### 3.9. Representative Curves Linearization

The representative load curves, obtained in the previous stage (Figure 12(a)), were linearized. The first operation in the linearization process was the search for the consumption limits of the four time slots and the minute of the day when they were produced. In Table 3, the extreme values of representative curve in the precrisis period are characterized. Then the percentage of margin between the limits was defined in the 25%. The value of this margin was selected because it obtained a better curve characterization.  Figure 12(b) shows the linearized load curves that resulted from the process containing the base date 10/02/2005.

**Figure 13:**
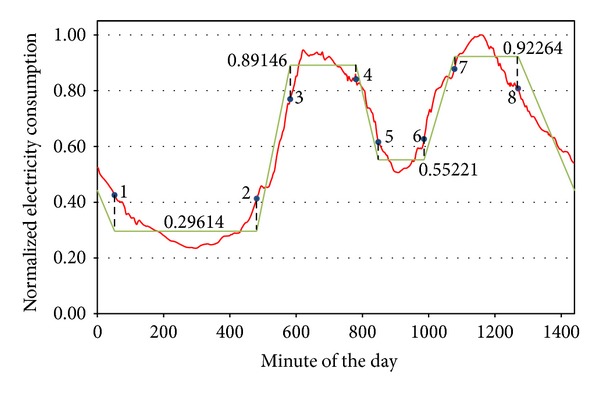
Linearization process of precrisis representative curve.

Using the limit values of Table 3 and following the process defined in the methodology, we calculated the new time borders and the normalized average consumption value of the daily slots. The result of these calculations, represented in Figure 13, was

- A: from minute 52 (Point 1) to 481 (2), with a value of 0.29614;
- B: from minute 582 (3) to 781 (4), with a value of 0.89146;
- C: from minute 848 (5) to 986 (6), with a value of 0.55221;
- D: from minute 1078 (7) to 1270 (8), with a value of 0.92264.

The A slot corresponds to the night period, the B corresponds to the morning, the C corresponds to the lunch time, and the D corresponds to the afternoon and evening. These time slots were adjusted according to Spain timetable.

This process ultimately yielded two linearized representative curves for each of the dates selected by the filters. Each curve was located in one of the two considered time periods (precrisis and crisis). These curves represent the daily EC of a winter day, from Monday to Thursday (filter f2), with air temperatures and EC near the base date values.

## 4. Results and Discussions

The method described for a specific date (10/02/2005 in the previous section) was applied to all the available dates (near 11 years). In order to conclusively analyse the EC changes during the economic crisis, statistic calculations were carried out using the linearized load curves.  Table 4 shows the results of the study about the duration of time slots. This table presents the percentage of days, for each month, with the duration of the time slots longer during the precrisis period than during the crisis period. We did not obtain values for March because the number of comparable dates was insufficient. Percentages less than 25% or greater than 75% were considered to be limit values in order to obtain conclusions.

When the maximum or minimum EC values before and during the crisis were analysed, we detected an increase in the minimum value in the night slot of July and August. The consumption during this slot was mainly residential (the consumption of the other consumers connected to the substation was not significant at those hours), and this increase was only perceived in the warmer months. Therefore, the larger consumption may be attributed to an increased use of air-conditioning systems due to an increase in the housing occupancy. During the crisis, citizens were more likely to stay at home in summer. In the morning slot of the cold months (January, February, and December), the maximum EC value was higher during the crisis, which may be attributed to consumers spending more time at home in the morning. The minimum value generally increased during the lunch slot, which may be attributed to an increased number of consumers eating lunch at home.

Observing the results of the slots duration, we can comment that the duration of the morning and afternoon and evening slots was very similar in the two periods. The night slot lasted longer during the crisis. This is evident because most of the months contained days that showed longer slots in the crisis period than in the precrisis period. For example, all the days in January and December have a longer night slot in the crisis period. The minimum EC was registered in this time slot. This change in the consumption behaviour was especially associated with the school activity months and the colder days, which can be attributed to an improvement in the energy efficiency of electric appliances and their reduced use. Electric appliances mainly refer to heating and lighting appliances here.

## 5. Conclusions

We have designed a new methodology for comparison of EC curves. The proposed methodology has been used to compare the EC curves of an urban low-voltage substation although it can be used for other types of substations. The comparable load curves were obtained using minute EC and daily outdoor temperature values. To realise the comparable load curves selection, the proximity on the calendar, day of the week, daylight time, and daily air temperature were considered, as well as the minute EC values. The resultant load curves in the two periods under study (precrisis and economic crisis) were compared. The studied parameters were the maximum or minimum EC values and the duration of the daily temporal slots.

The comparison of the linearized representative curves in the precrisis and the crisis periods shows significant differences in the duration of the time slots and in the corresponding maximum and minimum EC values. These differences can be attributed to a change in the customers behaviour.

The proposed methodology and the results obtained in its application in the load curves can be very valuable information for agents related to buying or selling of electric energy. Agents, as authorities and regulatory agencies, can use this information in order to establish the electricity tariffs taking into account social aspects. Other agents, as local distributors, can obtain information about the change in the customers behavior and so use it to establish their business strategies. This methodology can also be used to study the effect on the consumers of new tariffs schemes, public campaigns on energy efficiency at home, and the introduction of new residential electric appliances.

## Figures and Tables

**Table 1:** Format of the temperatures file (°C). Fragment of year 2002.

Date	Year	Day_week	Day_year	*t*_average_	*t*_maximun_	*t*_minimun_
2002/01/01	2002	11	1	5.2	5.3	5.2
2002/01/02	2002	4	2	5.1	5.2	5.1
2002/01/03	2002	5	3	5.9	6	5.9
2002/01/04	2002	6	4	7	7.1	7
2002/01/05	2002	7	5	7.1	7.2	7.1
2002/01/06	2002	11	6	7.4	7.5	7.3
⋮						
2002/06/07	2002	26	158	12.3	17.9	7.3
2002/06/08	2002	27	159	12.8	18.0	9.8
2002/06/09	2002	21	160	15.2	22.0	9.3

**Table 2:** Fragment of the thermal distances (°C) file corresponding to February 10, 2005.

Base date	Day type	Year02 day 14	Year02 day 13	Year02 day 12	Year02 day 11	Year02 day 10	Year02 day 9	Year02 day 8	Year02 day 7	Year02 day 6	Year02 day 5	Year02 day 4
09/02/2005	4	10.011	7.179	9.185	5.523	4.139	4.833	1.543	4.337	7.199	4.007	2.983
10/02/2005	5	5.63	7.457	5.167	5.123	3.847	2.025	2.166	6.638	1.838	3.444	6.644
11/02/2005	6	4.483	3.629	5.552	1.884	4.258	2.249	4.478	2.352	3.885	6.515	4.641
12/02/2005	7	2.759	5.953	3.164	7.259	6.547	1.453	6.646	5.324	5.742	2.832	3.226
13/02/2005	1	3.844	2.744	5.702	6.498	2.335	6.508	3.801	3.809	2.245	5.881	5.053
14/02/2005	2	7.228	4.738	8.515	8.44	8.242	4.625	4.657	7.115	12.684	10.588	10.024
15/02/2005	3	7.265	11.058	10.526	10.777	7.057	6.298	9.142	14.807	13.037	12.494	13.525

**Table 3:** Limit values of the representative curve in the precrisis period.

	Consumption limits	Minute
A minimum	0.235104	297
B maximum	0.945946	621
C minimum	0.50647	907
D maximum	1	1158

**Table 4:** Percentage of days where the time slots lasted longer during the precrisis period than during the crisis.

	% night	% morning	% lunch	% afternoon
January	**0.00**	35.00	40.00	52.50
February	14.58	27.08	31.25	29.17
March	No results	No results	No results	No results
April	25.00	34.38	50.00	53.13
May	6.06	12.12	33.33	66.67
June	16.67	33.33	58.33	33.33
July	80.00	43.33	43.33	**100.00**
August	44.44	30.56	50.00	52.78
September	23.53	47.06	52.94	82.35
October	7.69	38.46	38.46	15.38
November	2.00	28.00	40.00	26.00
December	**0.00**	50.00	46.67	26.67

Annual	19.58	33.73	43.67	49.70
